# Curriculum Transformation: From Didactic to Competency-Based Programs in Pharmaceutical Medicine

**DOI:** 10.3389/fphar.2019.00278

**Published:** 2019-03-21

**Authors:** Orin Chisholm

**Affiliations:** Pharmaceutical Medicine Unit, Department of Pharmacology, School of Medical Sciences, UNSW Sydney, Sydney, NSW, Australia

**Keywords:** pharmaceutical medicine, postgraduate education, curriculum development, online education, professional development, competency-based learning

## Abstract

As the complexity of the pharmaceutical industry increases and with the current disruptive forces affecting it, there is an increasing need for suitably-qualified personnel. Universities must respond to the need for graduates with the appropriate skills and knowledge to enable the transformation and future growth of this industry. Restructuring educational offerings to focus on graduate attributes, such as analytical and critical thinking, collaboration and problem solving, creativity, flexibility and self-direction in the context of the pharmaceutical industry facilitates the changes needed for future growth and viability. This paper discusses the process of program transformation to enable the development of graduates who can respond to these challenges in the pharmaceutical industry.

## Introduction

It is said that we now live in a VUCA world: volatile, uncertain, complex and ambiguous; a scenario that can be applied across a number of industries including healthcare and education (Casey, 2014). Many of the disruptive forces affecting the healthcare industry today are also impacting education: digital disruption, the increase in flows of commerce, trade and people across national borders, and the rise of economic power in the East (Dobbs et al., 2015). The increasing voice of the patient and the rise of patient-centricity in drug development are mirrored by the increasing student voice and student focus in education. These factors are all leading to the emergence of new business models in the pharmaceutical and wider healthcare industries, and in education (McCluskey and Winter, 2012; Crow and Dabars, 2015; Downs and Velamuri, 2016; Godman et al., 2018). Both sectors need to become more agile and adaptable to meet the needs of their customers. In educating professionals entering or working in the pharmaceutical industry, we therefore need to design programs that can equip graduates to meet these challenges. To this end, the Master of Pharmaceutical Medicine program at UNSW Sydney has been transformed from a didactic, instructivist model to a fully online delivery model fostering collaboration and connections between students, enabling them to develop learning networks which will support them as they evolve in their careers. This paper will explore the transformation and renewal processes undertaken so far in transitioning this program to a professional -competency-based program that equips students with the skills, knowledge and connections needed to meet the rapidly changing needs of their chosen careers in pharmaceutical medicine, which is the medical science discipline concerned with the discovery, pre-clinical and clinical development, evaluation, registration, safety monitoring, reimbursement and medical aspects of medicines used for therapeutic treatment (Daniels, 2011).

There is a growing need for qualified personnel to meet the demands of the pharmaceutical industry as therapeutics become more complex, the clinical and regulatory environments become more complicated, pricing environments become more restrictive and demands from patients and healthcare providers for education about therapeutics increase. With increasing pressures on the current operating model for large pharmaceutical companies and an increase in outsourcing critical functions, such as early stage research and development, clinical trials and regulatory affairs in Australia, there is an increased need for higher education institutions to deliver adequately trained employees who can hit the road running and provide immediate value to their employers (Ansell, 2013; Rasmussen and Foss, 2014). In 2017, the Australian federal government released the National Innovation and Science Agenda with the view to equip Australia for a transition from a low-value to a high-value manufacturing and services economy. One of the key pillars in this agenda is the need for skills and competencies development. In addition, the government has established a Medical Technology and Pharmaceuticals industry growth centre (MTPConnect) to drive the development of these industries. A central theme within the strategic agenda for this organization is skills development. The Commonwealth Scientific and Industrial Research Organization (CSIRO) has reviewed the future workforce growth required in this area. They have identified skills shortages in the sector and emphasized the need for greater training and education in areas such as clinical trials, advanced manufacturing, regulatory affairs and therapeutic product development (Commonwealth of Australia Department of the Prime Minister Cabinet, 2016; MTPConnect, 2016; CSIRO Futures, 2017). A more recent survey has identified a shortage in regulatory scientists in Australia (Cowles et al., 2017). Finally, the Medical Science Liaison Society has identified a 20% expected growth rate in the Medical Science Liaison role (a field-based medical affairs function) in the pharmaceutical industry over the next couple of years^1^.

At the same time, higher education is undergoing a transformation in the way education is being delivered worldwide, with an emphasis on digital transformation and skills development to ensure graduates are equipped to meet the significant disruptive forces affecting many industries and societies today and into the future (Barber et al., 2013; Adams Becker et al., 2018). This transformation includes the significant growth in online education, which has been steadily growing over the past 14 years, driven recently by the Massive Open Online Course (MOOC) phenomenon (Kumar et al., 2017; Seaman et al., 2018). Part of this growth has been due to the transformation of old-style distance education programs into fully online programs, taking advantage of the affordances of the digital environment to foster collaborative, connected and immersive learning environment for students (Dabbagh and Kitsantas, 2012). Pedagogy has developed to focus on 21st-century skills development in digital and information literacy, collaboration and teamwork, analytical and problem-solving skills, flexibility and adaptability, creative and critical thinking, leadership, initiative and self-direction (Beetham and Sharpe, 2013).

With this context in mind, it was an opportune time to review the Master of Medical Science in Drug Development program, which had been provided by UNSW Sydney for 20 years. UNSW Sydney is one of Australia’s largest research-intensive universities with a student body of over 50,000 and 6,000 staff. At the same time as this review of the program, the university was implementing an ambitious strategy that includes a commitment to academic excellence by embracing digital innovation, the formation of communities, inspired teaching methodologies, incorporating students as partners in educational reform, and closing the loop with greater feedback and dialogue between all partners in the educational endeavor.

The Drug Development program had originally been designed in an era focusing on didactic, instructivist education with an emphasis on knowledge delivery using early models of distance education that relied heavily on large volumes of paper-based notes, individual assignments and summative exam assessments (Anderson and Dron, 2011). To facilitate connections, students were required to attend the university campus four times per year for intensive weekend schools, which was a costly and inequitable activity, especially for students based around the country and overseas.

## Curriculum Transformation

**FIGURE 1:**
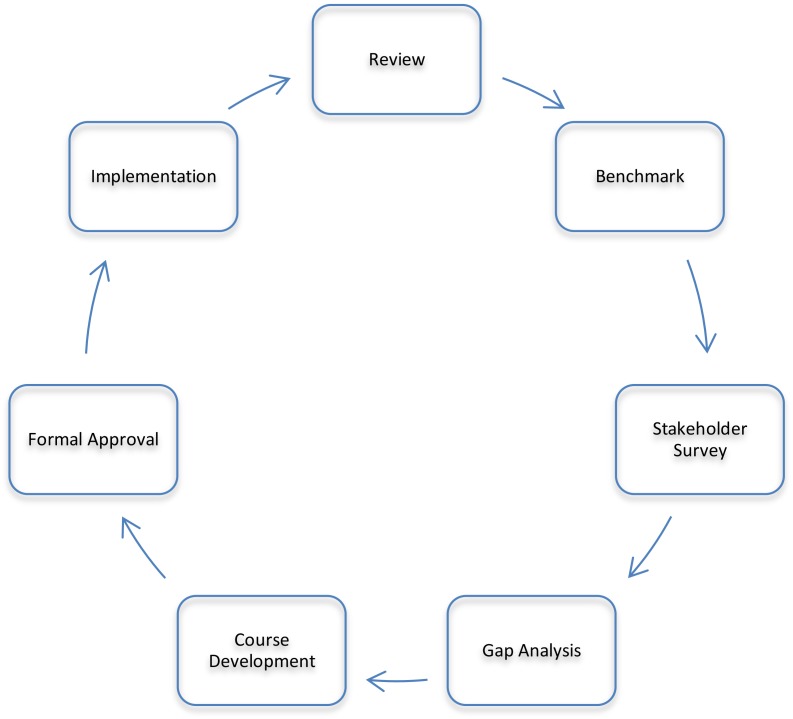
Curriculum redesign process.

To ensure the program remains relevant to the rapidly-changing pharmaceutical environment, a process was developed to systematically review and restructure the program based on sound pedagogical principles and the local pharmaceutical industry requirements (Chisholm, 2017). This process is shown in Figure 1. The process consisted of a thorough review of the existing program, benchmarking against similar programs, a stakeholder survey and review, a gap analysis, development of new courses, revision and rationalization of existing courses, formal approval by the university, and implementation followed by an evaluation of how the revised program is delivering against the plan. This process is cyclical and has the potential to be applied to any program of study needing updating.

The starting point for the development of the revised program was to articulate the mission of the program, which is to produce graduates with the knowledge and skills to make a meaningful contribution to medicines research, development and access, working across the pharmaceutical industry, academia and government, with the goal of improving the health and wellbeing of the community. Therefore, the program was aimed at people wanting to pursue a range of career possibilities in pharmaceutical drug discovery and development, medical device development, preclinical testing, clinical trials, drug safety and pharmacovigilance, regulatory affairs, medical and scientific communications, medical affairs, product compliance or health technology assessment within industry, regulatory agencies, academia or government health departments. Students enter the program with a variety of prior qualifications and knowledge: healthcare professionals (physicians, pharmacists, nurses, veterinarians), scientists with undergraduate qualifications, other Masters’ degrees or PhDs (in pharmacology, medicinal chemistry, pharmaceutical sciences, molecular biology, biochemistry, immunology, microbiology, medical science, biological science) and a small proportion with arts, psychology or law degrees.

Besides a thorough review of the existing content, a benchmark analysis of existing programs was conducted to identify areas of commonality and consensus. Stakeholders were surveyed to refine the elements needed for the revised program, identify gaps in coverage of the existing program and look at the future directions of the industry, the skills and knowledge that would be required by graduates in their careers (Allen and Chisholm, 2018). A gap analysis using all of this information helped to frame the program level learning outcomes (PLOs) for the revised program. Additionally, the PLOs were required to meet the Australian Qualifications Framework (Australian Qualifications Framework Council, 2013). The PLOs that were developed are listed in Table 1. They also reflect those outlined by the International Federation of Associations of Pharmaceutical Physicians (IFAPP) and Pharmatrain (Silva et al., 2013; Payton et al., 2013; Kerpel-Fronius et al., 2015; Dubois et al., 2016; Criscuolo, 2017^2^), but with the added competences of collaborative team work, development of digital and information literacy skills, an emphasis on development of a personal ethical framework, a global outlook and a thorough understanding of the pricing of new therapies. The emerging importance of the medical affairs role was recognized, and a new course was developed within the restructured program to address this gap in education (Moss et al., 2015). The revised program now consists of the following courses: introduction to the pharmaceutical industry, clinical trials, clinical trials management, Australian regulatory affairs, international regulatory affairs, pharmacovigilance, medical affairs, health technology assessment in Australia, advanced health technology assessment, pharmaceutics, general therapeutics, cancer therapeutics and an internship course. All courses and the program have been formally approved by the university and implemented since 2016. The revised program structure and course descriptions are available in the university handbook^3^, ^4^. Previous versions of the handbook are also accessible on the university website. Prior to this year, each course ran for one semester of approximately 14 weeks duration. From 2019, each course will run for a term of approximately 10 weeks duration, as the university has moved to a trimester academic calendar. The amount of content, activities and assessment tasks remain unchanged in this transition.

**Table 1:** Program-level learning outcomes.

Advanced disciplinary knowledge and practice	Graduates will be able to demonstrate an advanced understanding of pharmaceutical medicine and the development process for new therapeutic products and apply their knowledge to new developments and approaches within this area.
Enquiry-based learning	Graduates will have the ability to ask the appropriate questions, find relevant information using their digital and information literacy skills and develop the required plans and documents to facilitate their contributions to the development and maintenance of therapeutic products.
Cognitive skills and critical thinking	Graduates will be able to understand, critically appraise and apply information and literature in the field of pharmaceutical medicine to inform development of new therapeutic products or strategies for success of new and existing products.
Communication, adaptive and interactional skills	Graduates will demonstrate the ability to effectively communicate complex, relevant subject matter relating to pharmaceutical medicine to diverse audiences. They will have the appropriate skills of flexibility and adaptability in working collaboratively with others in teams to achieve specified outcomes in a time-bound environment. Graduates will show leadership and initiative in areas of their focus within pharmaceutical medicine.
Global outlook	Graduates will have a thorough knowledge and understanding of the global arena in which therapeutic products are developed, regulated, priced and marketed.
Ethics	Graduates will reflect on and critique the role of ethics in the therapeutics industry and develop a personal ethical framework for working within the area of pharmaceutical medicine.


The redevelopment of courses within the program followed a competency-based model that included bringing together teams of academics, industry experts, alumni and current students, as described by Drago et al., 2016. Pedagogical theories on how new knowledge is integrated during the learning process and how collaborative learning enhances this formed the foundation for course design (Vygotsky, 1980; Gersick et al., 2000; Siemens, 2005; Lim and Honey, 2006; Rajagopal et al., 2012; Yuan and Kim, 2014). Therefore, the activities and assessments that students now undertake require them to utilize the new knowledge and skills they are developing to produce an assessible item (Koohang et al., 2009). After all, “It’s not what we do but what students do that’s the important thing” (Biggs and Tang, 2011). Another powerful method for encouraging students to integrate their new knowledge and skills is by reflective practice and all courses contain a reflective practice element (Schön, 1983; Boud and Molloy, 2013; Naraynassamy, 2015).

In their courses, students form communities of practice with the others in their course by contributing to the weekly discussion forums and working together in teams to complete assessment tasks and activities designed into the curriculum (Wenger et al., 2002). However, students also reach out beyond these groups to other students, alumni and teachers of their courses, as well as their professional networks, including their work colleagues. Students are encouraged to form these wider connections during their learning to enhance and cement the knowledge and skills they are developing in each course. All of these connections form part of the student’s personal learning network and contribute to their continued learning beyond their graduation, becoming life-long learners (Moses and Duin, 2015; Wald, 2015).

The other important element in redesigning programs is to ensure there is constructive alignment between the program-level learning outcomes, the course-level learning outcomes, the activities and assessments that students undertake (Biggs and Tang, 2011; Fink, 2013). Courses are delivered online using the Moodle learning management system (Conde et al., 2014). The course design on Moodle follows the principles of the RASE system (resources, activities, support, evaluation) developed by Mirriahi et al. (2015). Authentic activities are designed into each course and include active learning and problem-based scenarios focussed on real life situations to develop problem-solving and critical thinking skills (Herrington et al., 2003). As an example, in a course on regulatory affairs, students are required to formulate an appropriate strategy for the registration of a new product, as one of the course-level learning outcomes that meets the program-level learning outcome of advanced disciplinary knowledge and practice. In order to meet this learning outcome, students have an assessment task where they are provided with a scenario and asked to prepare a report to their managing director outlining the regulatory strategy that they will pursue to obtain registration of the product described in the scenario. The scenario is a complex one where students need to integrate their understanding of two separate regulatory systems in Australia in order to determine the most efficient pathway forward. Aligning the activity, course and program outcomes like this ensures that the students meet them and graduate with the skills and experience required to undertake their roles in the pharmaceutical industry. As a further example of the evolution of assessment tasks in the program, see Table 2 below, which describes the assessment tasks for this course before and after the restructure. Detailed marking rubrics have been developed for each assessment task to ensure consistency of marking and to help students understand the requirements of the task. Assessment tasks are designed around real-life activities that students will conduct in their work such as writing reports, reviewing a dossier of information for a regulatory submission, or designing a clinical trial protocol.

**Table 2:** Assessment tasks for one course before and after restructure.

PHAR9101 (2013)	PHAR9101 (2019)
Quiz 10%	Group project – online wiki 40%
Individual responses to online exercises 10%	Individual essay assignment 30%
Individual assignment (3 parts) 40%	Online discussion forum contributions 15%
Invigilated final exam 40%	Individual reflective journal 15%


The only synchronous activities scheduled are the webinars, which are held once a week, in the evenings, to facilitate student participation. Course moderators and tutors often present a lecture and discuss case studies with students during these sessions, and these conversations continue in asynchronous discussion forums which are moderated by the tutors. In several courses, students are required to give presentations in the webinars and lead discussion on their presentations. The internship course provides hands-on experience for students wanting to deepen their practice in a particular area, such as regulatory affairs, medical affairs, clinical research, etc. Placements are available to students in companies, with the regulator or academia. This aspect of the program enables students to develop level four competencies on Miller’s pyramid by documenting performance integrated into practice (Miller, 1990).

The program is delivered fully online using the affordances of the Moodle learning management system and the internet (Harasim, 2000). Drivers for transition to an online delivery mode included:

• The ability to maintain currency of information in a fast-evolving industry
• A desire to improve collaboration between students located distantly from each other (an essential skill for working in the multinational pharmaceutical industry)
• A desire to increase self-evaluation and peer-evaluation (Boud and Molloy, 2013)
• The need to equip students with 21st century skills: digital and information literacy, critical and creative thinking, problem-solving, persistence, collaboration and teamwork, flexibility and adaptability, leadership, initiative and self-direction (McCluskey and Winter, 2012).

## Impact of the Program Transformation

The impact of changes to the program has been measured initially by both qualitative and quantitative methods. Success of the program transformation process has been and continues to be evaluated using student satisfaction feedback, analysis of their active engagement in the learning activities, assessment grades and employment outcomes. To date, there has been an increase in student engagement and collaboration throughout the program. This is seen with the increased numbers of posts to online discussion forums, which have increased from approximately three posts/student/course to 12 posts/student/course between 2015 and 2017. As well, students are responding to each other’s posts and developing deep conversations around the course topics and scenarios provided in these discussion forums. Furthermore, students value the opportunity to engage and learn with each other: “*The Discussion Forums continue to offer great learning opportunities and the chance to bond with other students”.* Students now undertake one group-based assessment task in each course throughout the program and this also fosters development of a sense of community, which is essential to the success of online learning programs (Gersick et al., 2000). These group-based tasks enable students to develop the networking and collaborative skills needed for a successful career in the pharmaceutical industry. The internship course will continue to be reviewed against Miller’s pyramid to capture competency in actionin the workplace.

Overall student satisfaction ratings across the program have increased from 4.3+/-1.2 to 5.1+/-0.4 (from a total score out of 6) between 2013 and 2017, providing evidence that students are seeing the benefit of the new alignment and learning and teaching style. Student grades have increased very slightly over the transition to a fully online program, showing consistency in learning even though student cohorts have changed over the years with slightly more now transitioning from another career. Academic staff teaching into the program have undertaken further postgraduate study in higher education. Industry- and government-based casual lecturers who currently teach on the program have significant experience with over 60% having appointments at director level or above in their own workplace. This greater expertise of all the teachers in the program is reflected by the higher student satisfaction ratings.

Finally, graduate employment levels are high for the program, with approximately 87% of graduates working in industry by their graduation (up from between 33 and 50% working in industry on entry to the program). In addition, approximately 80% of those who were working in industry at entry to the program managed to transition roles into the one they wanted or gained a promotion by graduation, including some who have taken promotions to positions based overseas. Further evaluation of the impact of the program transformation is planned and will include long-term follow-up of students after graduation, particularly reviewing transfer of learning to the workplace and career progression (Miller, 1990; Kirkpatrick and Kirkpatrick, 2006). This review may also result in further changes to the program as it evolves in-line with the evolution of the practice of pharmaceutical medicine.

## Conclusion

In conclusion, the Master of Pharmaceutical Medicine program at UNSW Sydney is now delivered as a fully online program using the affordances of the digital environment to develop capable graduates ready to take on challenging careers in the pharmaceutical industry. Their capabilities are developed through authentic learning experiences involving active experiential learning using problem-based scenarios focused on real-life situations to develop problem-solving and critical thinking skills. Additionally, students are learning in a connected environment that mirrors many of the interactions and situations they will face in their workplace, as we become more globally connected and work increasingly in digitally connected teams. As the working world changes, so too must the roles of educators and higher education continue to evolve. Thus, the delivery mode of the program is agile and able to be quickly adapted to meet future industry needs for skilled graduates, with Faculty acting not only as knowledge-experts, but as learning facilitators, for our students.

## Data Availability

The datasets generated for this study are available on request to the corresponding author.

## Author Contributions

OC was responsible for the concept and writing of this paper and takes full accountability for the content.

## Conflict of Interest Statement

The author declares that the research was conducted in the absence of any commercial or financial relationships that could be construed as a potential conflict of interest. The handling Editor DD and reviewers PS and HS declared their involvement as co-editors in the Research Topic, and confirm the absence of any other collaboration.

## Footnotes

1 http://www.themsls.org/

2 https://www.pharmatrain.eu/guidelines.php

3 https://www.handbook.unsw.edu.au/postgraduate/programs/2019/9370?q=9370\&ct=all

4 https://www.handbook.unsw.edu.au/Pharmacology/browse?sa=c2ce43204f0f5b00eeb3eb4f0310c77f
